# Tidal volume in animal models of hemorrhagic and endotoxic shock

**DOI:** 10.3892/etm.2013.969

**Published:** 2013-02-20

**Authors:** FEI LIU, TAO MA, ZHI LIU

**Affiliations:** Department of Emergency, The First Affiliated Hospital of China Medical University, Shenyang 110001, P.R. China

**Keywords:** multiple organ injury, mechanical ventilation, hemorrhagic shock, tidal volume

## Abstract

The aim of this study was to examine the characteristics of lung, kidney and small intestine injury caused by early resuscitation from hemorrhagic shock (HS) and endotoxic shock (ES) when ventilating with different tidal volumes (Vts). The study also considered the determination of the appropriate Vt for use in mechanical ventilation (MV) during treatment for shock. Resuscitated rabbits were ventilated with varying Vts for 120 min following 60 min of HS or ES. The histopathology, ultrastructure and apoptotic index (AI) of the lung, kidney and small intestine were then measured. Organs from the high-Vt groups (VT=12–15 ml/kg) showed the highest pathological scores (PSs; P<0.05). For HS, the renal PS and AI of the HS-M group (Vt=8–10 ml/kg)were lower than those of the HS-L group (Vt=4–6 ml/kg) and the lung PS and AI of the HS-C (control) group were lower compared with those of the HS-M group. For ES, the lung PS of the ES-L group was lower compared with that of the ES-M group (Vt=8–10 ml/kg) and the lung AI of the ES-C (control) group was lower compared with that of the ES-L group (Vt=4–6 ml/kg). When ventilated with the same Vt, ES resulted in higher PSs in the lung and intestine and a lower renal PS (P<0.05) than HS. MV was not recommended for either shock type. When it was necessary for MV to be applied, low Vt (4–6 ml/kg) protected the lung in ES. Moderate Vt (8–10 ml/kg) may be relatively safe to use for HS.

## Introduction

Shock is a common intensive clinical event that leaves patients vulnerable to multiple organ distress syndrome (MODS), particularly in the case of septic shock (1,2). Mechanical ventilation (MV) is an important measure used in shock care (3,4); however, studies concerning the use of MV in shock resuscitation are very rare. Since the strategy of using small tidal volumes (Vts) for acute respiratory distress syndrome (ARDS) patients has been proven and widely accepted in the Cochrane research (5–7), a judgment of the appropriate Vt for shock has yet to be established.

When considering protective mechanical strategy, protection of the body as a whole is necessary. Previous studies have suggested a possible link between MV and the development of MODS (8–11). Therefore, the present study aimed to compare multiple organ injury caused by hemorrhagic shock (HS) and endotoxic shock (ES) under MV at different Vts. This study also aimed to provide an approach for determining the appropriate Vt during early resuscitation from the two types of shock.

## Materials and methods

### Animal preparation

A total of 64 New Zealand white rabbits weighing 2.5–3.2 kg (provided by the Animal Laboratory of China Medical University) were randomly divided into eight groups (n=8 each group; Table I).

The rabbits were anesthetized using 300 g/l urethane (3–4 ml/kg intravenously) via a marginal ear vein and tracheotomy was then performed. The right carotid arteries were catheterized for blood pressure monitoring (HP monitor D-1034; HP, Boeblingen, Germany) and blood withdrawal for HS induction. The right regular veins were catheterized for re-infusion of the shed blood, isotonic saline and dopamine for shock resuscitation. After 15 min of stabilization breathing without MV, the artery blood gas was measured (using an AVL Omni 3 blood gas analyzer; AVL, Graz, Austria). The selected index was pH 7.30–7.40, PaO_2_>80 mmHg and PaCO_2_=35–45 mmHg. During the entire animal experiment, the central body temperature was kept between 38 and 39°C with the use of an electric blanket. All animal work was conducted in accordance with NIH guidelines (NIH Pub. No. 85-23, revised 1996) and was approved by Animal Care and Use Committee of China Medical University (Shenyang, China).

### HS, ES and resuscitation model

For the HS groups, HS was induced by withdrawing blood from the right carotid in aliquots of 2 ml/min to reduce the mean arterial pressure (MAP) to 40 mmHg over 20 min. The MAP was maintained at 38–42 mmHg over the next 40 min by withdrawing or re-injecting blood as required. Afterwards, the shed blood and isotonic saline were infused intravenously at an infusion speed of 2 ml/min to maintain the MAP at 65–80 mmHg and the heart rate (HR) at 160–240 bpm for 120 min. The blood was anti-coagulated with 400 U/kg heparin and the syringes containing the shed blood were placed on a horizontal rotator at 37°C at 170 × g (12). Dopamine infusions were used to reach the required MAP and HR when the saline infusion speed was >20 ml/kg/h or HR <160 bpm, for fast saline infusion.

For the ES groups, ES was induced via the intravenous infusion of 1 mg/kg endotoxin (L-2880 from *E. coli* sero-type 055:B5; Sigma, St. Louis, MO, USA) into the rabbits at a speed of 0.1 mg/min. MAP was reduced to 38–42 mmHg over ∼20 min and maintained at 65–80 mmHg for the next 40 min. Afterwards, 400 U/kg heparin was infused at a speed of 20 U/min/kg for 20 min. Isotonic saline and dopamine were also infused to maintain the MAP at 65–80 mmHg and HR at 160–240 bpm over 60 min.

Dopamine was administered to zero, five, one and two animals in groups HS-C, HS-L, HS-M and HS-H, respectively. All animals in the ES groups were administered dopamine. There were no significant differences in the amounts of dopamine administered among the four ES groups (data not shown).

### MV

The 60-min shock period without MV was followed by a 120-min resuscitation period during which the animals of the ventilated groups were myo-relaxed via peritoneal injection of vecuronium bromide (0.2 mg/kg/h). The animals were also ventilated (Bear 1000/es respirator; Viasys Healthcare, Palm Springs, CA, USA) with a specific Vt, FiO_2_ 40%, HR 40 bpm, and positive end-expiratory pressure (PEEP) 0 cmH_2_O. The animals of the control groups were breathing oxygen independently (via catheter, 5 l/min) without the administration of myo-relaxin.

### Hemodynamic data and arterial blood gas

After the 120-min resuscitation period, MAP, HR and the amounts of resuscitation fluid and dopamine were measured. Arterial blood gas was measured simultaneously, immediately after sampling.

### Tissue, plasma and tissue preparation

At the end of the experiment, the animals were sacrificed using an intravenous overdose of sodium pentobarbital. The abdomen and chest of the animals were opened. Surgical silk was tied around the left pulmonary artery and vein.

Small tissue fragments (5×5×5 mm^3^) were removed from the left dorsal lobe and stored in 2.5% glutaraldehyde for transmission electron microscopy (EM) analysis. The right lobes from the right pulmonary artery were fixed by instilling 0–4°C saline for 30 min and 4% buffered paraformaldehyde for 15 min, at a constant pressure of 15 cmH_2_O (10). The small fragments of the instilled right dorsal were then removed and stored in 10% formalin for light microscopy (LM) histology and terminal deoxynucleotidyl transferase dUTP nick end labeling (TUNEL) staining analysis.

Small fragments of the cortex and outer medulla of the right kidney (5×5×5 mm^3^) and small intestine (10×10 mm^2^) (10 cm below the Treitz ligament) were harvested and stored in 2.5% glutaraldehyde for EM analysis, and in 10% formalin for LM and cell death (TUNEL) analysis.

### Histology, EM and in situ cell death detection

The histological sections (5 *μ*m) of the lung, right kidney and small intestine were stained with hematoxylin and eosin (H&E). Histological assessment was performed by a professional histopathologist, blinded for the treatment, using the assessment methods of Brégeon *et al* (13), Paller *et al* (14) and Chiu *et al* (15). TUNEL staining was performed using an *in situ* cell death detection kit (Roche, Mannheim, Germany) according to the manufacturer’s instructions. The apoptotic index (AI) was the mean number of apoptotic cells per 100 cells in five fields of view (×400). The ultrastructure changes were determined by transmission EM (×8,000). Professional pathologists, who were blinded for the treatment, measured the AI and observed the ultrastructural changes.

### Statistical analysis

SPSS software for Windows (version 11.5) was used for statistical analysis. The data are presented as the mean ± SEM and tested for normal distribution using the Kolmogorov-Smirnov test. The data of the four groups per shock model were compared using one-way analysis of variance (ANOVA). The Student-Newman-Keuls post hoc test was used to compare significant ANOVA results among the groups. Comparisons among the data of the two shock groups using the same Vt (or control groups) were performed using a Student’s t-test. P<0.05 was considered to indicate a statistically significant difference.

## Results

### Blood gas

**Figure 1:**
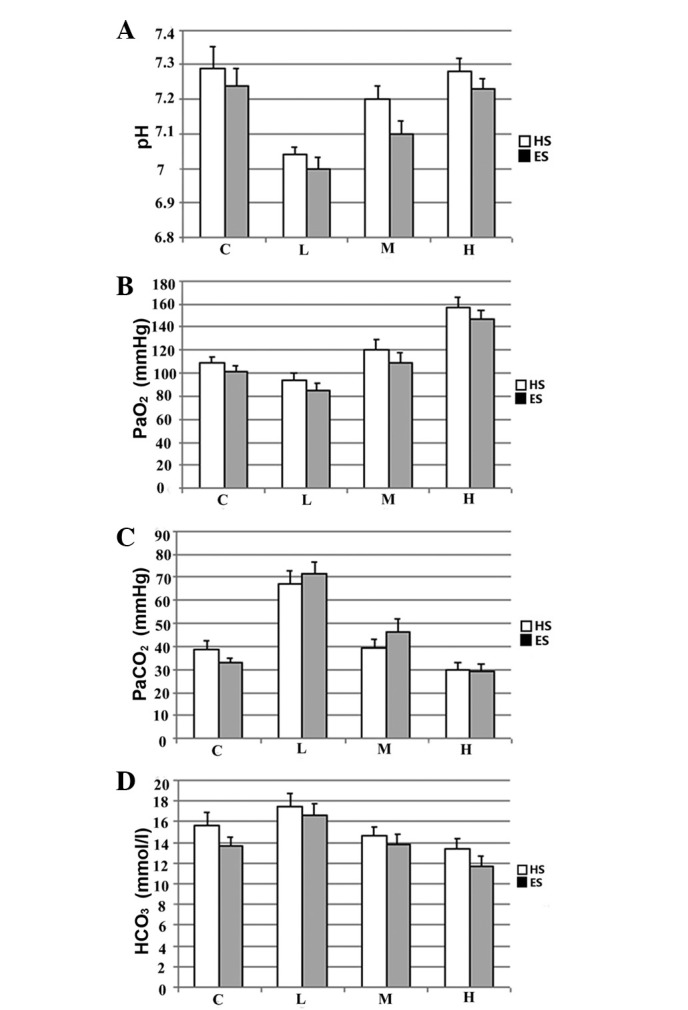
Blood gas. (A) pH; (B) PaO_2_ (mmHg); (C) PaCO_2_ (mmHg); (D) HCO_3_ (mmol/l). HS, hemorrhagic shock; ES, endotoxic shock; C, control; L, low Vt; Vt, tidal volume.

Following the 120-min resuscitation with ventilation at a specific Vt, arterial PaO_2_ and pH were significantly increased. By contrast, PaCO_2_ significantly decreased with increasing Vt. When ventilated with the same Vt, no significant differences were identified in the blood gas data between the rabbits in the HS and ES groups (Fig. 1).

### Pathological changes in the lung, kidney and small intestine

For HS and ES, the high-Vt groups showed more severe injuries of the lung, kidney and small intestine than the other Vt groups.

### Lung

**Figure 2:**
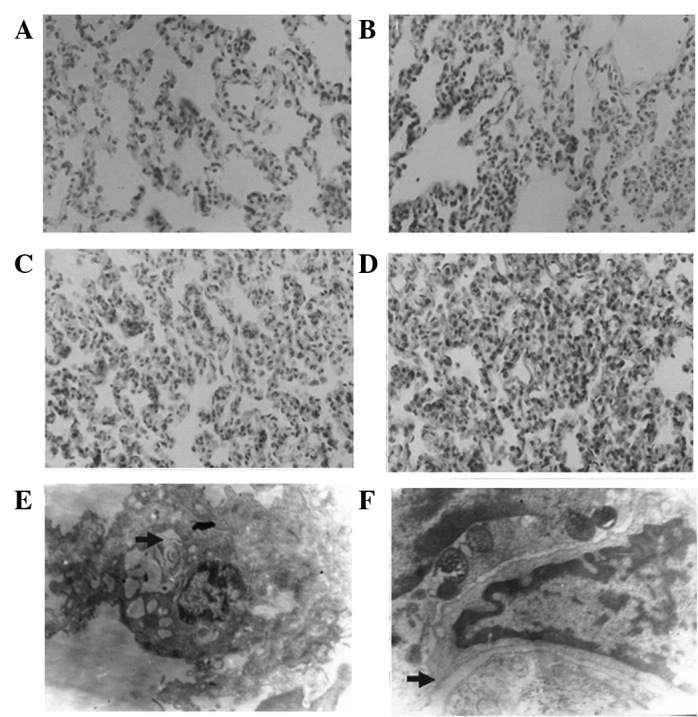
Pathological changes of lung. (A) Lung histology of group HS-M by LM (H&E, ×400): alveolar septum widened; (B) lung histology of group HS-H by LM (H&E, ×400): the injury was more severe than that in group HS-M: alveolar septum widened and alveoli collapsed; (C) lung histology of group ES-M by LM (H&E, ×400): the injury was more severe than that in group HS-M: alveolar septum widened, alveoli collapsed and intra-alveolar space was effusive; (D) lung histology of group ES-H by LM (H&E, ×400): the injury was more severe than that in groups ES-M and HS-H: alveoli collapse and intra-alveolar space effusion were more severe; (E) diminished electron density of luminal bodies in HS (EM, ×8,000). (F) Widened intercellular junction in ES (EM, ×8,000). EM, electron microscopy; H&E, hematoxylin and eosin; LM, light microscopy; HS, hemorrhagic shock; ES, endotoxic shock; M, moderate Vt; H, high Vt; Vt, tidal volume.

In the rabbits with HS, changes to the lungs included widening of the alveolar septum, as observed by LM (H&E, ×400) and the diminution of the electron density of luminal bodies, as observed by EM (×8,000). In the rabbits with ES, LM and EM showed intra-alveolar effusion and widening of the intercellular junction, respectively (Fig. 2).

### Kidney

**Figure 3:**
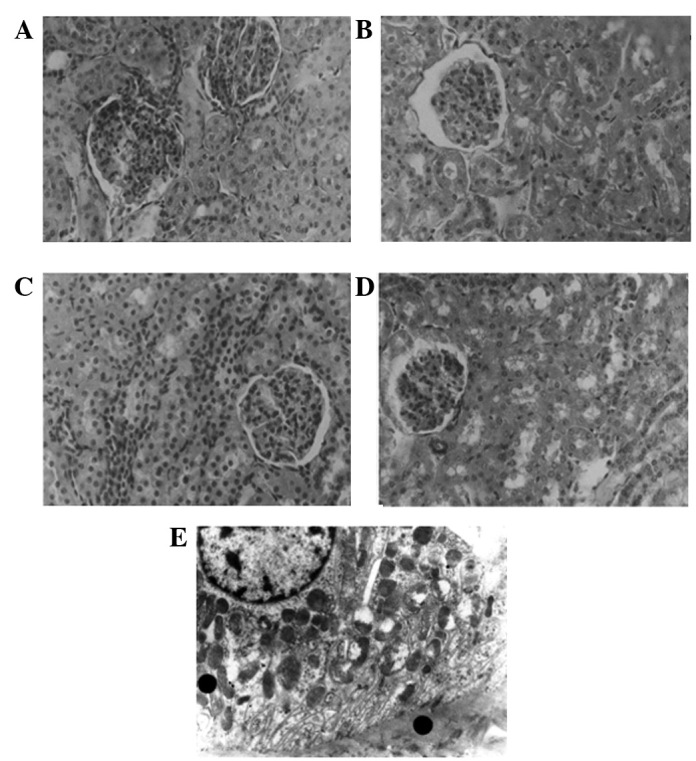
Pathological changes of kidney. (A) Renal histology of group HS-L by LM (H&E, ×400): renal interstitial edema; (B) renal histology of group HS-H by LM (H&E, ×400): renal tubular cell injury was more severe than that of groups HS-L and ES-H, the interstitial edema was significant; (C) renal histology of group ES-L by LM (H&E, ×400): renal tubular cell injury was slighter than that of group HS-L; (D) renal histology of group ES-H by LM (H&E, ×400): renal tubular cell injury was more severe than that of group ES-L, numerous cell fragments formed and blocked the tubular lumen; (E) renal histology of group ES-M under EM (×8,000): the mitochondria of the tubular epithelial cells were swollen and certain crista were ruptured. EM, electron microscopy; H&E, hematoxylin and eosin; LM, light microscopy; HS, hemorrhagic shock; ES, endotoxic shock; L, low Vt; M, moderate Vt; H, high Vt; Vt, tidal volume.

In the kidneys of the rabbits with HS, the interstitial edema was more marked, as observed by LM, and the mitochondria of the tubular epithelium cells showed more severe damage when examined by EM (Fig. 3).

### Small intestine

We have previously reported the pathological changes in the small intestine (16). The intercellular junction of the epithelia in the rabbits with ES was more severe, as observed by EM.

### Pathological scores (PSs) and AI

**Figure 4:**
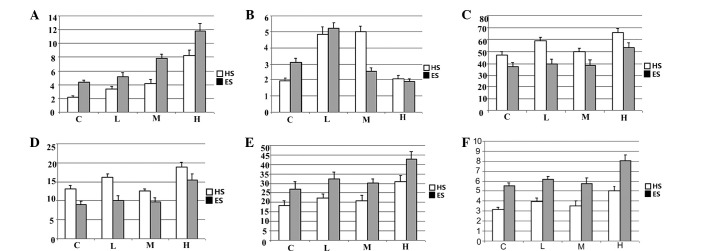
Pathological score and apoptotic index. (A) pathological score of lung; (B) apoptotic index of lung; (C) pathological score of kidney; (D) apoptotic index of kidney; (E) pathological score of small intestine; (F) apoptotic index of small intestine. C, control; L, low Vt; M,moderate Vt; H, high Vt; Vt, tidal volume.

For HS and ES, the PSs and AIs of the organs in the high-Vt groups (HS-H and ES-H) were higher than those of the organs from other groups, with the exception of the lung, which had a lower AI (Fig. 4).

For HS, a comparison between the HS-L and HS-M groups showed that the renal PS and AI of the HS-M group were lower, whereas no statistically significant differences were observed between the groups with regard to the PS and AI of the lung and intestine. A comparison between the HS-C and HS-M groups demonstrated that lung PS and AI of the HS-C group were lower, whereas no statistically significant differences were noted with regard to the PS and AI of the kidney and intestine (Fig. 4).

For ES, a comparison between the ES-L and ES-M groups revealed that lung PS of the ES-L group was lower. The PS and AI of the kidney and intestine showed no statistically significant differences between the two groups. A comparison between the ES-C and ES-L groups indicated that the lung AI of the ES-C group was lower.

In rabbits ventilated with the same Vt, the organs in the HS groups showed higher PSs and the small intestine and kidney in the HS groups showed higher AIs. Lung AI was affected differently: the control groups had lower lung AIs and the high-Vt groups also had lower lung AIs than the other Vt groups.

## Discussion

The correlation between MV and distal organ injury has been addressed in a series of studies. Imai *et al* noted that MV is associated with distal organ damage in rabbits with lung injury caused by hydrochloric acid during intra-tracheal administration (8). The injurious effects of MV on the extra-pulmonary organs of mice with experimental *S. aureus* pneumonia were also demonstrated in the study by Dhanireddy *et al* (9). O’Mahony *et al* demonstrated non-injurious MV strategies using a conventional Vt of 10 ml/kg. Interaction with endotoxemia induced via the intra-peritoneal injection of *E. coli* LPS in mice enhanced pro-inflammatory mechanisms in the lungs and promoted extra-pulmonary end-organ injury (10). A study by Wolthuis *et al* further indicated that ventilator-associated lung injury (VALI) occurs despite the absence of a priming pulmonary insult and despite the use of non-injurious ventilator settings (11).

Occasionally, MV is required for hemorrhagic or septic shock patients in intensive care units, who are susceptible to acute lung injury (ALI), ARDS and MODS, but do not meet the criteria for ALI or ARDS. We proposed that despite the lack of significant evidence of lung injury in such cases, protective MV should be administered to avoid the development of lung and distal organ injury. Thus, in the present study, the ventilated rabbit models of HS and ES were developed.

The results of the current study showed that high Vt (12–15 ml/kg) may increase the pathological injury of the lung and distal organs when the lung injury does not meet the criteria for ALI/ARDS. High Vt is harmful during early resuscitation from HS and ES.

The initial purpose of the present study was to determine a suitable ventilation Vt. Our data indicated that MV was injurious and should be avoided if possible during early resuscitation from shock. Compared with the ventilated groups, the control groups for the two types of shock showed slightly less pulmonary injury and extra-pulmonary organ injury. Data from Wolthuis relating to non-injurious ventilation (11) and commentary from Ng *et al* concerning gene expression changes following ventilation (17) also indicated that ‘non-injurious’ ventilation does not necessarily mean ‘protective’. Our data indicated that MV was injurious and should be avoided if possible during early resuscitation from shock.

Reduction of PEEP is the most important ventilation strategy component affecting hemodynamic stability. Zero or even negative end-expiratory pressure is safe for HS (18–20), which is why we used a PEEP of 0 cmH_2_O during the resuscitation period. Herff *et al* reported that reducing the mean airway pressure by decreasing Vt had less effect than 0 cmH_2_O PEEP on cardiopulmonary function and survival (20). The effects of varying Vts on HS observed in our study were as follows. Given constant blood pressure and fluid infusion, the HS-C, HS-L, HS-M and HS-H groups contained zero, five, one and two animals, respectively, that required treatment with dopamine. The greater need for dopamine in the HS-L group may be attributed to mixed acidemia (respiratory and metabolic acidosis). Ventilating with 4–6 ml/kg Vt in HS may cause circulation instability. The HS-M and HS-L groups had similar lung and small intestine PSs, but the latter group had a higher renal PS and AI. Increased dopamine use may aggravate renal injury (21). These results indicated that should MV be required during early resuscitation from HS, a moderate Vt of 8–10 ml/kg may be relatively safe.

By contrast, differences in Vt seemed to have less effect on hemodynamic stability during ES, given that the sub-groups had similar blood pressures and used similar amounts of dopamine and fluid infusion. The ES-L and ES-M groups showed similar renal and intestinal PSs and AIs, and the former showed a lower lung PS. If it is not possible to avoid MV and the noxious stimuli that lead to shock which are also important triggers for ARDS, a low Vt of 4–6 ml/kg is encouraged during early resuscitation from ES.

The different recommended Vts should be based on the physiological differences between the two types of shock. In HS, the blood capacity is absolutely deficient whereas in ES, the blood capacity is relatively deficient. Reperfusion injury during resuscitation may be more severe in HS (22). Endotoxins may cause more damage in ES. Studies have shown that endotoxins damage the intercellular junctions in the lung (23) and intestine (24,25) and may increase the susceptibility of lung tissue to VALI (10,26). The present study reports additional differences between ES and HS, which hitherto had not been directly compared. In HS, pathological injury to, and apoptosis in, the lung and small intestine were slighter, but the renal pathological injury and apoptosis were more severe than in ES. The required dopamine dosage during ES resuscitation was also higher than in HS resuscitation. Certain studies have shown that the use of dopamine may improve alveolar fluid re-absorption (27,28), intestinal microcirculation and renal β-2-microglobulin excretion (21), as well as reduce intestinal inflammation (29). The results of the current study indicate that the use of dopamine may alleviate lung and intestinal injury and aggravate renal injury.

The decreased lung apoptosis and increased lung PS observed in the high-Vt groups of rabbits were in stark contrast with the effects observed in the kidney and small intestine. The current data were in agreement with the study by Imai *et al*, in which rabbits were exposed to two bouts of acid aspiration and VALI (8) with the hypothesis that milder injury may result in a greater degree of apoptosis in the lungs.

The blood gas data in the current study showed no significant difference between HS and ES resuscitation using the same Vt. This result, which may be due to the time of observation, also indicated that ventilation was an important factor affecting blood gas during early resuscitation. Further comparative studies are necessary to better understand and improve the treatment of the two types of shock.

The blood gas data of the high-Vt groups indicated excessive ventilation. During intensive care, efforts are made to improve blood gas, oxygen saturation point and artery oxygen pressure. However, our study identified no association between higher artery oxygen pressure and reduced organ injury, reinforcing the standpoint of certain scholars regarding permissive hypoxia (30). It may be better to balance the oxygen supply to the organs against the protection of the lungs. Extracorporeal CO_2_ removal and extracorporeal membrane oxygenation may provide improved rest for the injured lung. The concept of complete rest is not a new approach, as it has long been applied in renal and liver replacement therapy, although the hemodynamic problems in the treatment of shock may prove more difficult.

The clinical Cochrane study of the effects of different Vts for the MV of shock patients may be more conclusive.

High Vt (12–15 ml/kg) was injurious to the lung and distal organs during HS and ES. MV was not recommended for either shock type, but when it was necessary for MV to be applied, a low Vt (4–6 ml/kg) protected the lung during ES. Moderate Vt (8–10 ml/kg) may be relatively safe for use during HS.

## Figures and Tables

**Table I:** Grouping of animals.

Group	HS	ES
Control	HS-C	ES-C
Low Vt (4–6 ml/kg)	HS-L	ES-L
Moderate Vt (8–10 ml/kg)	HS-M	ES-M
High Vt (12–15 ml/kg)	HS-H	ES-H

HS, hemorrhagic shock; ES, endotoxic shock; Vt, tidal volume.
